# Cdk5/p35 functions as a crucial regulator of spatial learning and memory

**DOI:** 10.1186/s13041-014-0082-x

**Published:** 2014-11-18

**Authors:** Tomohide Mishiba, Mika Tanaka, Naoki Mita, Xiaojuan He, Kodai Sasamoto, Shigeyoshi Itohara, Toshio Ohshima

**Affiliations:** Laboratory for Molecular Brain Science, Department of Life Science and Medical Bioscience, Waseda University, 2-2 Wakamatsu-cho, , Shinjuku-ku Tokyo, 162-8480 Japan; Laboratory for Behavioral Genetics, Brain Science Institute, RIKEN, Saitama, 351-0198 Japan

**Keywords:** Spatial learning, Memory, Kinase, Synaptic plasticity, Hippocampus

## Abstract

**Background:**

Cyclin-dependent kinase 5 (Cdk5), which is activated by binding to p35 or p39, is involved in synaptic plasticity and affects learning and memory formation. In Cdk5 knockout (KO) mice and p35 KO mice, brain development is severely impaired because neuronal migration is impaired and lamination is disrupted. To avoid these developmental confounders, we generated inducible CreER-p35 conditional (cKO) mice to study the role of Cdk5/p35 in higher brain function.

**Results:**

CreER-p35 cKO mice exhibited spatial learning and memory impairments and reduced anxiety-like behavior. These phenotypes resulted from a decrease in the dendritic spine density of CA1 pyramidal neurons and defective long-term depression induction in the hippocampus.

**Conclusions:**

Taken together, our findings reveal that Cdk5/p35 regulates spatial learning and memory, implicating Cdk5/p35 as a therapeutic target in neurological disorders.

**Electronic supplementary material:**

The online version of this article (doi:10.1186/s13041-014-0082-x) contains supplementary material, which is available to authorized users.

## Background

Cyclin-dependent kinase 5 (Cdk5) is a serine/threonine kinase that is abundant in neuronal cells and is activated by complexing with p35 or p39. Recent studies have demonstrated that Cdk5 is critically involved in synaptic plasticity, a cellular basis of memory formation [1], in addition to its function in neuronal development [2-5]. At presynaptic terminals, Cdk5 regulates neurotransmitter release via phosphorylation of presynaptic proteins such as synapsin I and N-type voltage-gated calcium channels [6,7]. Alternatively, at postsynaptic dendritic spines, Cdk5 phosphorylates postsynaptic proteins such as postsynaptic density protein 95 (PSD-95) [8], NMDA receptor subunit NR2A [9], protein phosphatase inhibitor-1 [10], dopamine- and cAMP-regulated neuronal phosphoprotein (DARPP-32) [11], and tropomysin-related kinase B (TrkB) [12]. Moreover, inducible Cdk5 conditional knockout (cKO) mice show enhanced synaptic plasticity and improved spatial learning and memory via an increase in synaptic NR2B subunits of NMDA receptors [13]. More recently, it was shown that disrupting long-term potentiation (LTP) and long-term depression (LTD) in the hippocampal CA1 of mice lacking Cdk5 results in the impairment of spatial learning and memory partly due to the collapse of cAMP signaling [14]. These two results, however, are likely secondary consequences of the loss of Cdk5 function as a scaffold protein [15] and a regulator of other signaling pathways, respectively. Therefore, the original functions of Cdk5 in synaptic plasticity and in the phosphorylation of synaptic proteins remain to be elucidated.

We have previously demonstrated an impairment of spatial learning and memory, and hippocampal LTD induction in p35 knockout (KO) mice. However, p35KO mice exhibit reversed cortical lamination of cerebral cortex and mild disorganization of cellular alignment of CA1 pyramidal neurons and dentate gyrus [3]. These histological abnormalities in the brain may confound these results [16]. To further investigate the role of Cdk5/p35 in higher brain function without histological abnormalities, we generated inducible p35 conditional knockout (cKO) mice, in which p35 is deleted in all cells by breeding p35-flox mice with CreER-mice [17]. We found that CreER-p35 cKO mice have defective spatial learning and memory, along with decreased anxiety-like behavior. We also showed reduced spine density of pyramidal neurons, reduced sensitivity to synaptic input, and impaired LTD induction in the hippocampal CA1. Biochemical analysis revealed no alteration of NR2B protein in the hippocampi of CreER-p35 cKO mice. These findings indicate that the kinase function of Cdk5/p35 is essential for normal synaptic function and spatial learning and memory.

## Results

### Reduction of p35 protein in the brains of p35-flox; CreER mice by oral administration of tamoxifen

**Figure 1:**
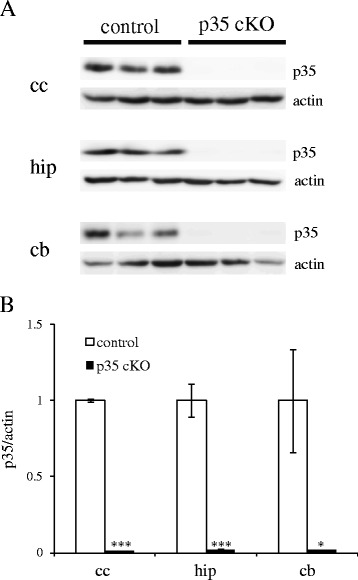
**Inducible p35 protein reduction in the CreER-p35 cKO mouse brain. (A, B)** Tamoxifen was administered orally to CreER-p35 cKO mice (black bars, p35 cKO) and p35-flox mice (white bars, control). 1 week after administration, p35 protein was significantly reduced in CreER-p35 cKO mice when compared with control. Unpaired *t-*test, *p < 0.05, ***p < 0.001.

To analyze the function of Cdk5/p35 in the adult mouse brain, we induced Cre activity by oral administration of tamoxifen at 6 months of age. We fed mice with 3 mg · 40 g^−1^ body weight tamoxifen for three days and analyzed protein levels of p35 in different brain regions of p35-flox; CreER mice after one week. As shown in Figure 1, we found a significant reduction of p35 protein levels in all brain regions examined when compared with tamoxifen-fed p35-flox mice (hereafter control mice). Therefore, we are able to use tamoxifen to induce conditional deletion of p35. Protein levels of Cdk5 in hippocampi of CreER-35 cKO mice were found comparable with those of control mice (Additional file 1: Figure S1).

### Normal locomotor activity and reduced anxiety-like behavior in CreER-p35 cKO mice

**Figure 2:**
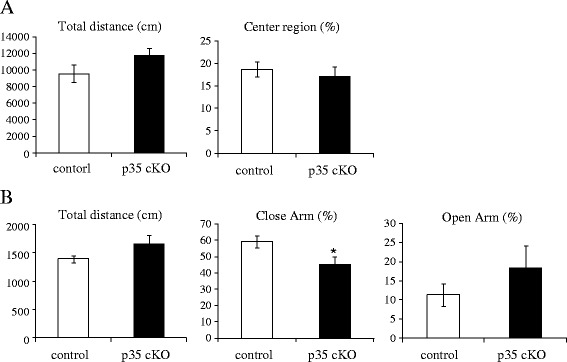
**Normal locomotor activity and reduced anxiety-like behavior in CreER-p35 cKO mice (A)** Total distance (cm) and percentage time spent in the center region of the open field in p35-flox (white bars, control, n =6) and CreER-p35 cKO mice (black bars, p35 cKO, n =8). No difference was observed between these. **(B)** Total distance (cm) and percentage time spent in closed and open arms in the elevated plus maze in p35-flox (white bars, control, n =6) and CreER-p35 cKO mice (black bars, p35 cKO, n =8). CreER-p35 cKO mice traveled a similar distance when compared with control and displayed less anxiety-like behavior. Error bars represent standard error of the mean (S.E.M.). Unpaired *t*-test, *p < 0.05.

To evaluate the role of Cdk5/p35 in higher brain function, we tested control and CreER-p35 cKO mice with behavioral tests. Using an open field test to investigate locomotor activity, we found no differences in total distance of horizontal movements and spending time in center region of the open field among the two genotypes (Figure 2A).

The anxiety-like behavior of CreER-p35 cKO mice was assessed with an elevated plus maze (Figure 2B). The total distance traveled by CreER-p35 cKO mice was a little longer than that by control mice. The time CreER-p35 cKO mice spent in the closed arm was significantly shorter than that in control mice, while CreER-p35 cKO mice remained in the open arm somewhat longer than control mice. These results suggest that CreER-p35 cKO mice have reduced anxiety-like behavior.

### Defective spatial learning and memory in CreER-p35 cKO mice

**Figure 3:**
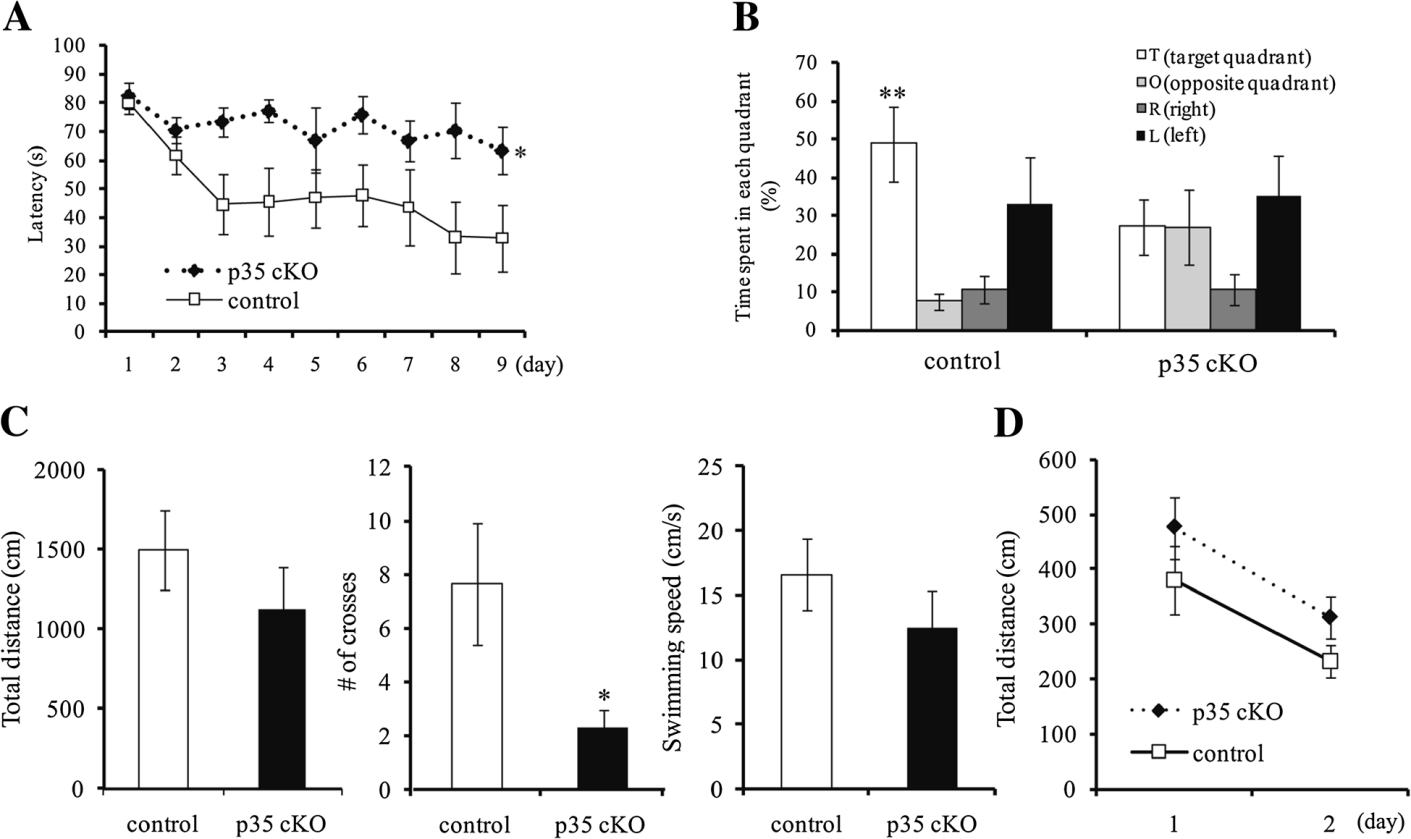
**Defective spatial learning and memory in CreER-p35 cKO mice. (A)** Latency (s) over 9 testing days in the Morris water maze in p35-flox (open square, control, n =6) and CreER-p35 cKO mice (closed rhombus, p35 cKO, n =7). The slope of the learning curve was significantly lower in CreER-p35 cKO mice when compared with control mice (mixed ANOVA test, p =0.027). **(B)** The probe test after the hidden platform task. Control mice spent longer in the target quadrant (T) than other quadrants (One-way ANOVA test, p = 0.0054), while CreER-p35 cKO mice did not. **(C)** Total distance (cm), the number of crosses over the region where the platform was formerly located, and swimming speed (cm/s) of the probe test, and total distance (cm) in CreER-p35 cKO and control mice in the visible platform task. CreER-p35 cKO mice crossed the region fewer times than the control mice, and swam as far and as fast as control mice (unpaired *t*-test, p = 0.034). **(D)** No difference was observed in the total distance travelled in the visible platform task between the two genotypes. Error bars represent S.E.M. Unpaired *t*-test, *p < 0.05, **p < 0.01.

In a previous study, p35 KO mice exhibited impaired spatial learning and memory, although these mice showed some deficits in brain development as well [16]. In order to distinguish between secondary effects due to altered brain development and clarify the function of Cdk5/p35 in spatial learning and memory, CreER-p35 cKO and control mice were analyzed with the Morris water maze (Figure 3). Mice swam to find the hidden platform and escape the water. On the first training day, the latency to reach the platform was almost the same between the two genotypes. During the training sessions for 9 days, the latency to reach the platform significantly decreased in control mice but not in CreER-p35 cKO mice (Figure 3A).

The platform was removed after the hidden platform task, and mice were allowed to swim freely in the probe test. The time spent in the target quadrant (where the platform had been formerly located) was significantly longer than the time spent in the other quadrants for control mice, while the time spent in the target quadrant was not different from the time spent in the other quadrants for CreER-p35 KO mice (Figure 3B). The number of times that CreER-p35 cKO mice crossed the region where the platform used to be located was significantly less than that by control mice (Figure 3C). CreER-p35 cKO mice swam almost as far and as fast as control mice (Figure 3C). In the visible platform test, however, the total distance swam by CreER-p35 cKO mice was approximately equal to that by the control mice (Figure 3D). These results suggest that spatial learning and memory are significantly impaired in CreER-p35 cKO mice.

### Reduction in dendritic spine density in CreER-p35 cKO mice

**Figure 4:**
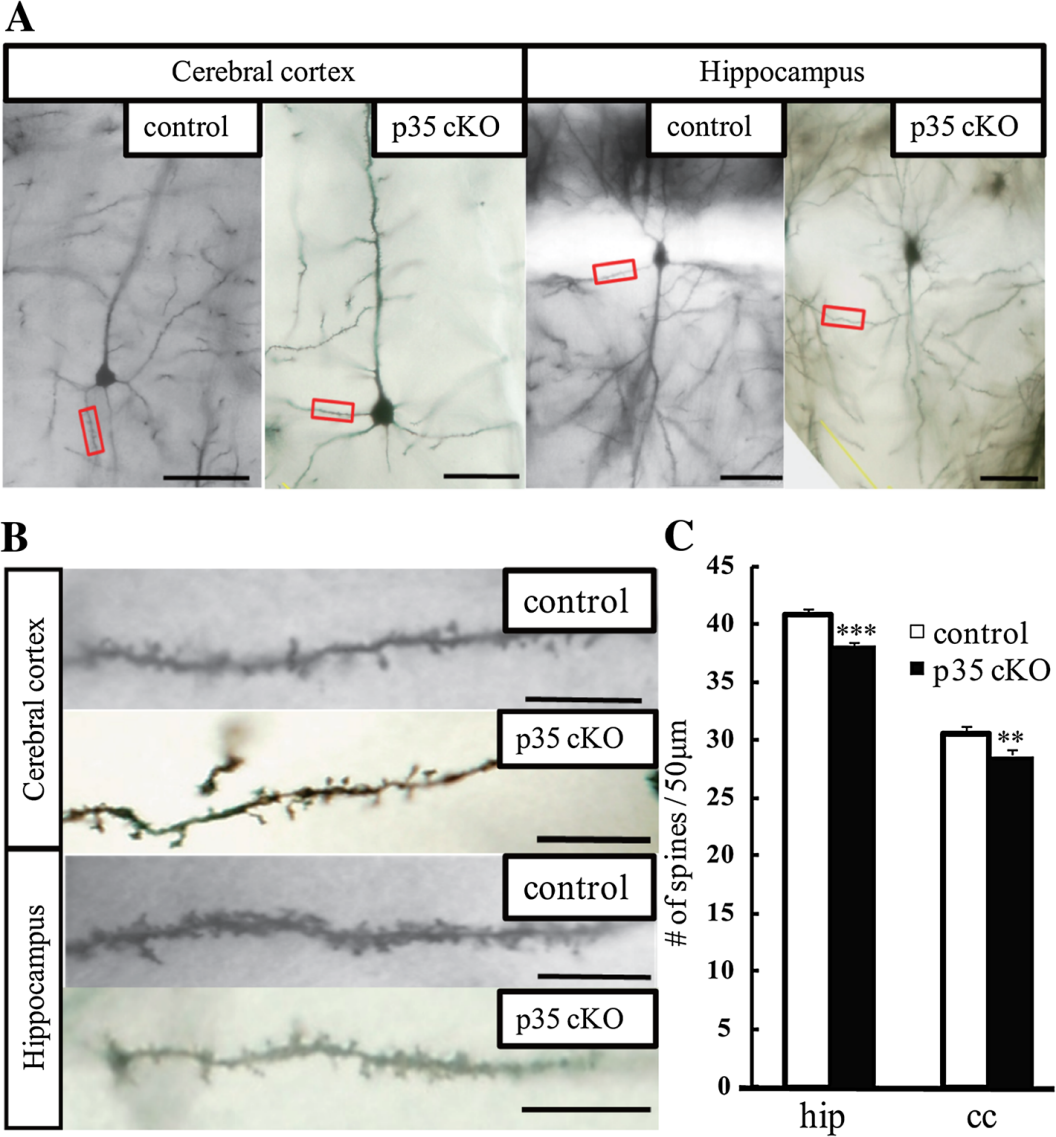
**Reduced dendritic spine density on pyramidal neurons in CreER-p35 cKO mice. (A)** Representative segments of the dendrites of pyramidal neurons in the cerebral cortex and hippocampus. Scale bar: 50 μm. **(B)** Higher magnified images of indicated areas of the dendrites of pyramidal neurons in the cerebral cortex and in the hippocampus. Scale bar: 10 μm. **(C)** Dendritic spine density in the cerebral cortex and hippocampus was significantly decreased in CreER-p35 cKO (p35 cKO, black bars) compared with p35-flox (control, white bars) mice. hip: hippocampus, cc: cerebral cortex. Error bars represent S.E.M. **p < 0.01, ***p < 0.001.

To clarify the mechanism underlying the impairment in spatial learning and memory, we studied the morphology of Golgi-stained pyramidal neurons in the hippocampal area CA1 and layer V in the cerebral cortex. We found that in CreER-p35 cKO mice, dendritic spine density on the apical dendrites of CA1 pyramidal neurons was significantly reduced. In addition, spine density on layer V basal dendrites in the cerebral cortex was significantly reduced when compared with control mice (Figure 4). Our findings suggest that the loss of dendritic spines on CA1 pyramidal neurons contributes to defective spatial learning and memory.

### Impaired synaptic properties in CreER-p35 cKO mice

**Figure 5:**
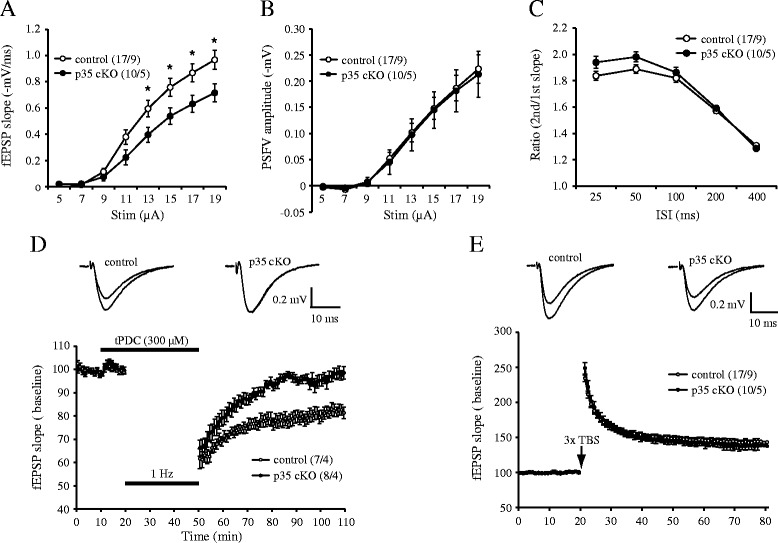
**Impaired basal synaptic transmission and LTD induction in CreER-p35 cKO mice. (A, B)** Input-output relationship. The slope of field excitatory postsynaptic potentials (fEPSPs) was significantly reduced in CreER-p35 cKO mice (control: n =17 slices [N =9 mice], p35 cKO: n =10 [N =5]) (A) with no change in presynaptic fiber volley amplitude (PSFV) (B) when compared with controls. *p < 0.05, unpaired *t*-test. **(C)** Synaptic facilitation in response to paired-pulses with an inter-stimulus interval (ISI) of 25–400 ms (control: n =17 [N =9], p35 cKO: n =10 [N =5]). **(D)** Long-term depression (LTD) induced by low-frequency stimulation (LFS, 1 Hz) combined with the application of the glutamate transporter blocker, tPDC. LTD was significantly impaired in CreER-p35 cKO mice when compared with controls. Insets are representative traces before and after LTD induction (control: n =7 [N =4], p35 cKO: n =8 [N =4]). **(E)** Long-term potentiation (LTP) induced by 3× theta-bust stimulation (3× TBS). LTP was comparable between genotypes. Insets are representative traces before and after LTP induction (control: n =17 [N =9], p35 cKO: n =10 [N =5]). Data represent mean ± S.E.M.

We examined the basal synaptic transmission of inducible CreER-p35 cKO mice. The slope of Shaffer collateral-CA1 field excitatory postsynaptic potentials (fEPSPs) was significantly reduced (*p* < 0.05) in CreER-p35 mice when compared with control mice (Figure 5A), with no change in presynaptic fiber volley amplitude (Figure 5B). We did not detect any significant differences in paired-pulse facilitation (Figure 5C), a form of short-term presynaptic plasticity. These results suggest that impaired synaptic transmission in CreER-p35 cKO was not caused by a presynaptic deficit.

To test whether loss of p35 affects synaptic plasticity, we next examined if there were any changes in hippocampal LTD in CreER-p35 cKO mice. LTD in adult mice was induced using low-frequency stimulation (LFS) combined with the application of the glutamate transporter inhibitor, tPDC [18]. To determine if this form of LTD is NMDAR-dependent, LTD was induced in the presence of the NMDA blocker, D-AP5. We found that LTD was significantly blocked by D-AP5, indicating that LTD is largely NMDAR-dependent (Additional file 2: Figure S2). We found that LTD induction was significantly impaired in CreER-p35 cKO mice when compared with control (Figure 5D; control, 82.1 ± 2.3%; p35 cKO, 98.2 ± 2.3%; unpaired *t*-test, p =0.0003). We next examined if LTP was affected in CreER-p35 cKO mice. Using 3× theta-burst stimulations (TBS), we found that there were no significant LTP differences between genotypes (Figure 5E). Thus, these results demonstrate that loss of p35 resulted in a reduction in the postsynaptic response and selectively affected NMDAR-mediated LTD.

### Biochemical analysis of hippocampal tissue from CreER-p35 cKO mice

**Figure 6:**
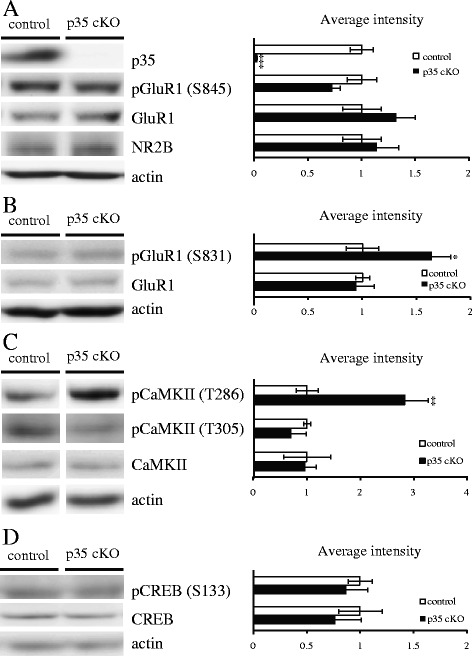
**Phosphorylation levels of GluR1 in CreER-p35 cKO mice.** Western blots of **(A)** p35, pGluR1 (S845), GluR1 and NR2B, **(B)** pGluR1 (S831) and GluR1 **(C)** pCaMKII (T286), pCaMKII (T305) and CaMKII, **(D)** pCREB (S133) and CREB in the hippocampi of CreER-p35 cKO mice (n =4) and control (n =4). pGluR1 (S845, S831) are normalized to GluR1, pCREB (S133) normalized to CREB, and others normalized to actin. The phosphorylation level of pGluR1 (S845) does not change notably, but pGluR1 (S831) is significantly increased in CreER-p35 cKO (p35 cKO) when compared with p35-flox (control) mice. In CreER-p35 cKO mice, the phosphorylation level of pCaMKII (T286) is significantly increased in comparison with p35-flox mice. The phosphorylation of pCREB (S133) is not altered between the two genotypes. Error bars represent S.E.M. Unpaired *t*-test, *p < 0.05, **p < 0.01, ***p < 0.001.

To investigate the molecular mechanisms underlying the deficits of spatial learning and memory, we performed biochemical analysis of postsynaptic proteins in the hippocampi of CreER-p35 cKO and control mice (Figure 6). GluR1-containing AMPA receptors and NR2B-containing NMDA receptors are involved in synaptic plasticity, leading to memory formation. In CreER-p35 cKO mice, protein levels of GluR1 and NR2B were not altered in the hippocampi when compared with that of control mice (Figure 6A). We also found that the phosphorylation level of pGluR1 (S845), a PKA phosphorylation site, was not altered, but levels of pGluR1 (S831), a CaMKII phosphorylation site, were dramatically elevated in the hippocampi of CreER-p35 cKO mice when compared with control mice (Figure 6A, B).

When CaMKII is activated, CaMKII autophosphorylates at T286. We found that the phosphorylation level of pCaMKII (T286) was significantly elevated in CreER-p35 cKO mice when compared with control mice (Figure 6C). We also analyzed phosphorylation level of CaMKII at T305, because previous study reported that activated CaMKII can promote either LTP or LTD depending on T305/T306 phosphorylation [18]. pCaMKII (T305) was reduced in CreER-p35 cKO mice, although the difference was not statistically significant (p =0.338).

CREB is one of the transcriptional factors implicated in memory formation that is controlled by cAMP signaling. We found no difference in the phosphorylation level of pCREB (S133) between CreER-p35 cKO and control mice (Figure 6D). These results suggest that the impaired spatial learning and memory and defective NMDAR-dependent LTD induction observed in CreER-p35 cKO mice are unrelated to cAMP signaling and the phosphorylation of CREB.

## Discussion

Our findings implicate Cdk5/p35 in spatial learning and memory, anxiety-like behavior, LTD induction, and the maintenance of spine density on hippocampal CA1 pyramidal neurons. These phenotypes of CreER-p35 cKO mice are similar to those of p35 KO mice [16] and CaMKIIcre-Cdk5 cKO mice [14], while they contradict findings from inducible Cdk5 cKO mice [13]. In inducible Cdk5 cKO mice, increased NR2B in the postsynaptic membrane may lead to unexpected phenotypes that include improved learning and memory [13]. Thus, the phenotype of inducible Cdk5 cKO mice did not represent a loss of Cdk5 kinase function [15]. CaMKIIcre-Cdk5 cKO mice exhibited impaired learning and memory with impaired cAMP signaling [14]. Since rolipram, an inhibitor of PDE4 and activator of cAMP signaling, rescued impaired learning and memory and hippocampal LTP, this phenotype is explained by impaired cAMP signaling [14]. In p35 KO mice, histological abnormalities make it uncertain that the observed phenotypes, including impaired spatial learning and memory, are caused by Cdk5/p35 loss-of-function of [16]. Our present study clearly supports the idea that Cdk5/p35 is required for spatial learning and memory [16].

Interestingly, defective LTD, but normal LTP in CA1, and the impairment of spatial learning and memory were also observed in p35 KO [16] and p35 cKO mice (Figure 5). In mice lacking GluN2B, also known as NR2B, pyramidal neurons in the cortex and hippocampal CA1 had defective NMDA-dependent LTD and impaired spatial learning and memory [19]. However, we found comparative protein levels of NR2B in the hippocampi of CreER-p35 cKO mice (Figure 6A). There are several explanations for impaired induction of LTD with the loss of Cdk5/p35. Cdk5 and calcineurin are implicated in the interaction of phosphatidylinositol 4-phosphate 5-kinase γ661 (PIP5Kγ661) and the clathrin adaptor protein complex AP-2, resulting in the internalization of AMPA receptors during LTD in the CA1 [20,21]. When the phosphorylation/dephosphorylation balance of PIP5Kγ661 is impaired, internalization of AMPA receptors in LTD will be compromised in the hippocampal CA1 of p35 cKO mice. The dephosphorylation of cofilin is required for NMDA receptor-dependent LTD in the CA1 [22]. Furthermore, the phosphorylation of cofilin is upregulated by the treatment of a Cdk5 inhibitor, suggesting that Cdk5 regulates NMDA receptor-dependent LTD in the hippocampal CA1 through cofilin [23]. In addition, Cdk5/p35 phosphorylates a number of postsynaptic proteins, including PSD-95 [8], Polo-like kinase 2 [24], NMDA receptors [9], and calcium channels. Reduced phosphorylation of these proteins may affect LTD induction. These possibilities should be tested in future experiments.

Cdk5 is involved in dendritic spine formation through the phosphorylation of ephexin1, WAVE1, and TrkB [12,25,26]. EphA4, which is activated by the phosphorylation of ephexin1 by Cdk5, is also necessary for synaptic plasticity in the amygdala [27]. Another study suggests that the phosphorylation of TrkB by Cdk5 is required for spatial memory and hippocampal LTP [12]. These studies reveal that spine formation, memory formation, and synaptic plasticity are closely related to each other. Our analysis of p35 cKO mice also identified reduced dendritic spines in pyramidal neurons in the cerebral cortex and hippocampal CA1 region (Figure 4). Therefore, defective synaptic plasticity, in addition to a reduction in spine density, is related to the behavioral changes that we observed in p35 cKO mice. It is also possible that the loss of dendritic spine densities in CA1 pyramidal neurons is one of the reasons why the input-output curve in CreER-p35 cKO mice is lower than that in control mice (Figure 5), as fewer dendritic spines on CA1 pyramidal neurons leads to decreased postsynaptic responses [28]. Reduction of dendritic spine densities of CA1 pyramidal neurons seems cell-autonomous because we observed similar reduction of spine density of these neurons in CA1-specific p35 cKO mice [29].

The GluR1 subunit is essential for LTP in the CA1 region of the adult hippocampus [30]. The phosphorylations of GluR1 at two sites, S831 by CaMKII and PKC and S845 by PKA, have been demonstrated to play a role in synaptic AMPA receptor regulation and synaptic plasticity [31,32]. Our biochemical analysis revealed elevated phosphorylation of GluR1 at S831, but not at S845, in hippocampal homogenates from CreER-p35cKO mice (Figure 6). We also found elevated phosphorylation of CaMKIIα at T286 (Figure 6), suggesting an activated state of CaMKIIα [33,34]. Previous studies have demonstrated the interaction between p35 and CaMKIIα [35] and elevated phosphorylation of CaMKIIα at T286 with the Cdk5 inhibitor, roscovitine [36]. Therefore, our results indicate that loss of p35 releases its inhibitory function on CaMKIIα and results in elevated phosphorylation of GluR1 at S831 (Figure 6). These biochemical changes may explain the lower LTP induction threshold observed in p35 KO mice [37]. Although the role of CaMKII activation in LTP have been extensively characterized, much less is known about its role in LTD. Pi *et al.* have reported that activated CaMKII can promote either LTP or LTD depending on T305/T306 phosphorylation [18]. Interestingly, we found a reduction of T305 phosphorylation of CaMKIIα in hippocampal homogenates from CreER-p35cKO mice (Figure 6). This phosphorylation site of CaMKIIα inhibits its kinase activity [38,39], suggesting an activated state of CaMKII. Phosphorylation of CaMKIIα at S567 has recently been implicated in LTD [40]. Thus, the relation between CaMKII activation and LTD induction warrants further investigation.

p25 is formed by the truncation of the N-terminus of p35 by calpain, and it activates Cdk5 by complexing with it to form Cdk5/p25. In previous studies, both overexpression and loss of p25 in the forebrain led to neuronal loss and neuroinflammation [4,41]. Thus, tight regulation of Cdk5 is necessary to further study higher brain function without neuronal loss and neuroinflammation. In order to exclude the possibility that neuronal loss and neuroinflammation are the causes of this phenotype, Cdk5 cKO mice that were generated previously should be re-evaluated from the viewpoint of neuronal loss and neuroinflammation. Importantly, in our study, loss of Cdk5/p35 results in a disturbance of synaptic plasticity without neuronal loss and neuroinflammation (Additional file 3: Figure S3).

In a recent study, the impairment of neurotransmitter release was observed in a forebrain-specific Cdk5 cKO, indicating defective presynaptic function with Cdk5 loss-of-function [42]. Therefore, we need to dissect the separate pre- and postsynaptic functions of Cdk5. As for postsynaptic function, we recently generated L7cre-p35 cKO mice, in which p35 is dramatically reduced in Purkinje cells in the cerebellum, and LTD induction was severely impaired in these Purkinje cells [43]. The investigation into the role of Cdk5/p35 in postsynaptic plasticity in the hippocampus by using CA1 specific cre mice will provide further insight into the role of Cdk5 on synaptic plasticity *in vivo*.

The phosphorylation of various substrates by Cdk5 crucially affects synaptic plasticity, spine morphology, and memory formation, ultimately influencing behavior. It remains to be elucidated how Cdk5 controls different forms of neural plasticity via its phosphorylation of diverse proteins. Our findings on the role of Cdk5/p35 in LTD in the hippocampus allow a better understanding of the molecular mechanisms underlying synaptic plasticity and memory formation.

## Conclusions

To investigate the role of Cdk5/p35 in higher brain function without histological abnormalities, we generated CreER p35 cKO mice. CreER-p35 cKO mice exhibited defective spatial learning and memory. Our analyses also revealed reduced spine density of pyramidal neurons, reduced sensitivity to synaptic input, and impaired LTD induction in hippocampal CA1. These results indicate that the kinase function of Cdk5/p35 is essential for normal synaptic function, and spatial learning and memory.

## Methods

### Materials

Tamoxifen (Sigma, MO) was dissolved in 99.9% methanol in 100 mg · ml^−1^, where DMSO was added (99.9% methanol: DMSO =5:2). 10 mg · ml^−1^ solution was made in corn oil (Sigma, MO).

### Generation of tamoxifen-induced p35 conditional KO mice

All experimental protocols were approved by Institutional Animal Care and Use Committees of Waseda University and RIKEN. Throughout the experimental procedures, all efforts were made to minimize the number of animals used and their suffering. Mice were fed *ad libitum* with standard laboratory chow and water in standard animal cages under a 12 h light/dark cycle.

p35-flox mice were generated as described on a C57BL/6 background [43]. CAGGCre-ER (CreER) mice [17] were obtained from Jackson laboratory (stock number 004682). By crossing p35flox/+ and CreER mice, p35-flox/flox; CreER mice were obtained. Cre activity was induced by oral administration of tamoxifen (3 mg · 40 g^−1^ body weight in corn oil) daily for three days. The resultant inducible p35 conditional KO mice were used as CreER-p35 cKO mice along with littermate controls that were given same tamoxifen treatment.

### Biochemical analysis

Hippocampi of the mice were collected from p35 cKO mice (n =4) and their p35-flox littermates (n =4) as controls at 6 months old. Western blotting analysis was conducted as previously described [44]. Primary antibodies used in present study are as follows; polyclonal antibody against p35 (C-19, 1:1000, Santa Cruz), anti-NR2B (1:1000, Cell signaling technology), anti-CREB (1:1000, Cell signaling technology), anti-pCREB (S133, 1:1000, Cell signaling technology), anti-GluR1 (1:1000, Cell signaling technology), anti-pGluR1 (S831/S845, 1:1000, Upstate), anti-CaMKII (1:1000, Chemicon), anti-pCaMKII (T286, 1:1000, Cell signaling technology), anti-pCaMKII (T305, 1:1000, Upstate) and anti-actin (1:1000, Sigma). Statistical analysis was conducted using the Student’s *t* test and mean ± standard error of the mean (S.E.M.) are shown on the graph. *p* < 0.05 was considered to be statistically significant.

### Spine density analysis

For modified Golgi–Cox staining, an FD Rapid Golgi Stain kit was used according to manufacturer’s instructions (FD NeuroTechnologies, MD). Stained slices were sectioned at a thickness of 150 μm. Pyramidal neurons of hippocampal CA1 and layer V in cerebral cortex from each mouse were selected and the number of spines in apical dendrites of the neurons in hippocampal CA1 and in basal dendrites of the neurons in layer V in the cerebral cortex were counted using a BX50 microscope (Olympus, Japan) with UPlanSApo 40× (NA =0.95) objective. In a typical experiment, over 2000 spines were counted on more than 50 dendritic segments in 25 neurons for each sample. Statistical analysis was conducted by the Student’s *t* test, and the mean ± S.E.M. are shown on the graph. *p* < 0.05 was considered to be statistically significant.

### Electrophysiology

#### Slice preparation

Electrophysiological experiments were conducted with 2–2.5 month-old male mice 2–5 weeks after oral administration of tamoxifen. Mice were deeply anesthetized with halothane (2-Bromo-2-chloro-1, 1, 1-trifluoroethane) and killed by decapitation. The brains were immediately removed and placed in an ice-cold cutting solution [containing (in mM) sucrose 200, KCl 3, NaH_2_PO_4_ 1, MgSO_4_ 10, CaCl_2_ 0.2, NaHCO_3_ 26, and d-glucose 10, saturated with 95% O_2_ and 5% CO_2_]. Transverse hippocampal slices (300 μm thick) were prepared from both hemispheres. The slices were incubated in the recording solution [containing (in mM) NaCl 120, KCl 3, NaH_2_PO_4_ 1, MgSO_4_ 1.25, CaCl_2_ 2.5, NaHCO_3_ 26, and d-glucose 10, saturated with 95% O_2_ and 5% CO_2_] at room temperature (25°C) for at least 1 h before recording.

#### Extracellular recordings

Field potential responses were recorded from the *stratum radiatum* area in the CA1, using an array of 64 planar microelectrodes (MED-P515A) arranged in an 8 × 8 pattern with an interelectrode spacing of 150 μm (Alpha Med Scientific Inc.). Schaffer collaterals were stimulated at 0.05 Hz by delivering biphasic current pulses (5–19 μA, 0.2 ms). Input-output relationships were measured as previously described [44]. For paired-pulse facilitation (PPF) and long-term potentiation (LTP), the stimulation intensities were chosen to produce an excitatory postsynaptic potential (fEPSP) with 30% amplitude of the maximal response. The PPF was induced by paired stimuli with increasing intervals from 25–400 ms. The facilitation ratio was calculated as described [45]. Long-term potentiation (LTP) was induced with 3 theta burst stimulations at 0.05 Hz after 20 min of stable baseline recording. Each theta burst consisted of 4 bursts (5 Hz), each of which consisted of 4 pulses (100 Hz). LTP was recorded for 60 min after induction, and the potentiation was evaluated with the average slope of the last 5 min recording (56–60 min). For long-term depression (LTD), the stimulation intensities were chosen to produce a fEPSP with 50% amplitude of the maximal response. LTD was induced by low frequency stimulation (1 Hz, 1800 s) in the presence of glutamate transporter inhibitor l-trans-pyrrolidine-2,4-dicarboxylic acid (tPDC, 300 μM) [19]. LTD was recorded for 60 min after induction, and the depression was evaluated with the average slope of the last 5 min recording (56–60 min). In a separate experiment examining if the LTD was NMDA receptor (NMDAR)-dependent, the NMDAR antagonist D-AP5 (50 μM) was co-applied with tPDC. Evoked field responses were, through a 0.1–10 kHz bandpass filter, recorded at a 20 kHz sampling rate and stored for off-line analysis. All data were acquired and analyzed with a MED64 Mobius (Alpha Med Scientific Inc.). All experiments were performed by an investigator blind to the mouse genotype. Student’s *t* test was used for statistical analysis.

### Behavioral analysis

All experiments were performed by an investigator blind to the mouse genotype; 3 mg · 40 g^−1^ body weight tamoxifen was given orally for three days to p35-flox and p35-flox; CreER male mice. After two weeks, 5–5.5 month old male mice were subjected to behavioral analysis as described below (p35-flox: n = 6, CreER-p35 cKO: n = 7). Student’s *t*-test and one-way/two-way ANOVAs were used for statistical analysis.

#### Open field

The locomotor activity of each mouse was assessed with an open field (60 × 60 cm) at 55 lx for 30 min. The mice were placed in the center of the area, and their horizontal movements were registered with a set of cameras and analyzed with TimeOFCR4 software [46].

#### Elevated plus maze test

The elevated plus maze apparatus was set at a height of 50 cm above the floor with 4 gray plexiglass arms, which were 2 opposite open arms (25 cm × 5 cm) and 2 opposite closed arms (25 cm × 5 cm × 15 cm). The apparatus was brightly illuminated (250 lx) at the surface of each arm. Every mouse was placed in the center of the apparatus; their movement was measured for 10 min, and analyzed with TimeEP1 software [46].

#### Morris water maze test

The Morris water maze test was performed as previously described with some modifications [47]. In this study, we used a pool with diameter of 150 cm. A circular platform (10 cm in diameter) submerged ~0.7 cm below the water surface was placed 37.5 cm from the center of the pool to either north or south quadrants for counterbalancing samples. Mice were given 4 trials per day for 7 consecutive days in the hidden platform task under brightly illuminated conditions (250 lx at the surface of maze). A randomly selected starting point along the rim of the maze was used for each of the four trials. A probe test was performed on day 8 after the acquisition session. In the probe test, the platform was removed from the tank and each mouse was allowed to swim for 60 s. On day 10, mice were tested in a visible platform task for 3 consecutive days. In the visible platform task, the platform was made visible by attaching a black cubic landmark to the platform. Mouse movement in the water maze was recorded by a video camera and analyzed using NIH image WM software (O’Hara & Co.).

### Statistics

The data are presented as mean ± S.E.M., unless otherwise noted. The statistical analyses were performed using the Student’s *t* test, one-way ANOVA, or two-way repeated measures ANOVA as described in the Results or in the Figure legends. *p* < 0.05 was considered statistically significant.

## Additional files

Additional file 1: Figure S1. No changes of Cdk5 protein levels in hippocampal homogenates from CreER-p35 cKO mice. Western blot analysis of Cdk5 protein in hippocampal homogenates from control and CreER-p35 cKO mice (p35 cKO) with anti-Cdk5 and anti-actin antibodies **(A)**. No difference was found in Cdk5 protein levels among two genotypes **(B)**. n = 4. Additional file 2: Figure S2. NMDA receptor-dependent LTD induction in the presence of tPDC. LTD induced by LFS in the presence of 300 μM tPDC was significantly blocked by application of D-AP5.Hippocampal slices from wild-type animals (C57BL/6 J) were used. Additional file 3: Figure S3. No neuro-inflammation in hippocampal CA1 in CreER-p35 cKO mice. Immunostaining of hippocampal sections of control and CreER-p35 cKO mice (p35 cKO) with Iba1 to observe activated microglia **(A)**, with GFAP to observe activated astrocytes **(B)** and with DAPI.

## Abbreviations

Cdk5: Cyclin-dependent kinase 5
KO: Knockout
cKO: Conditional knockout
LTP: Long-term potentiation
LTD: Long-term depression
P: Postnatal day
PPF: Paired-pulse facilitation
EPSP: Excitatory postsynaptic potential
LFS: Low-frequency stimulation
TBS: Theta-burst stimulation

## References

[1] Fischer A, Sananbenesi F, Pang PT, Lu B, Tsai LH (2005). Opposing roles of transient and prolonged expression of p25 in synaptic plasticity and hippocampus-dependent memory. Neuron.

[2] Ohshima T, Ward JM, Huh CG, Longenecker G, Veeranna, Pant HC, Brady RO, Martins LJ, Kulkarni AB (1996). Targeted disruption of the cyclin-dependent kinase 5 gene results in abnormal corticogenesis, neuronal pathology and perinatal death. Proc Natl Acad Sci U S A.

[3] Ohshima T, Gilmore EC, Longenecker G, Jacobowitz DM, Brady RO, Herrup K, Kulkarni AB (1999). Migration Defects of *cdk5*-/- Neurons in the developing cerebellum is cell autonomous. J Neurosci.

[4] Takahashi S, Ohshima T, Hirasawa M, Pareek TK, Bugge TH, Morozov A, Fujieda K, Brady RO, Kulkarni AB (2010). Conditional deletion of neuronal Cyclin-dependent kinase 5 in developing forebrain results in microglial activation and neurodegeneration. Am J Pathol.

[5] Kumazawa A, Mita N, Hirasawa M, Adachi T, Suzuki H, Shafeghat N, Kulkarni AB, Mikoshiba K, Inoue T, Ohshima T (2013). Cyclin-dependent kinase 5 is required for normal cerebellar development. Mol Cell Neurosci.

[6] Matsubara M, Kusubata M, Ishiguroi K, Uchidai T, Titani K, Taniguchi H (1996). Site-specific phosphorylation of synapsin I by mitogen-activated protein kinase and Cdk5 and its effects on physiological functions. J Biol Chem.

[7] Su SC, Seo J, Pan JQ, Samuels BA, Rudenko A, Ericsson M, Neve RL, Yue DT, Tsai LH (2012). Regulation of N-type voltage-gated calcium channels and presynaptic function by Cyclin-dependent kinase 5. Neuron.

[8] Morabito MA, Sheng M, Tsai L (2004). Cyclin-dependent kinase 5 phosphorylates the N-terminal domain of the postsynaptic density protein PSD-95 in neurons. J Neurosci.

[9] Li BS, Sun MK, Zhang L, Takahashi S, Ma W, Vinadei L, Kulkarni AB, Brady RO, Pant HC (2001). Regulation of NMDA receptors by cyclin-dependent kinase-5. Proc Natl Acad Sci U S A.

[10] Bibb JA, Nishi A, O’Callaghani JP, Ule J, Lan M, Snyder GL, Horiuchi A, Saito T, Hisanaga S, Czernik AJ, Nairn AC, Greengard P (2001). Phosphorylation of protein phosphatase inhibitor-1 by Cdk5. J Biol Chem.

[11] Bibb JA, Snyder GL, Nishi A, Yan Z, Meijer L, Fienberg AA, Tsai LH, Kwon YT, Giraultk J, Czernik AJ, Huganir RL, Hemmings HC, Nairn AC, Greengard P (1999). Phosphorylation of DARPP-32 by Cdk5 modulates dopamine signaling in neurons. Nature.

[12] Lai KO, Wong ASL, Cheung MC, Xu P, Liang Z, Lok KC, Xie H, Palko ME, Yung WH, Tessarollo L, Cheung ZH, Ip NY (2012). TrkB phosphorylation by Cdk5 is required for activity-dependent structural plasticity and spatial memory. Nature Neurosci.

[13] Hawasli AH, Benavides DR, Nguyen C, Kansy JW, Hayashi K, Chambon P, Greengard P, Powell CM, Cooper DC, Bibb JA (2007). Cyclin-dependent kinase 5 governs learning and synaptic plasticity via control of NMDAR degradation. Nature Neurosci.

[14] Guan J, Su SC, Gao J, Joseph N, Xie Z, Zhou Y, Durak O, Zhang L, Zhu JJ, Karl RC, Steven AC, Tsai LH (2011). Cdk5 is required for memory function and hippocampal plasticity via the cAMP signaling pathway. PLoS ONE.

[15] Hawasli AH, Bibb JA (2007). Alternative roles for Cdk5 in learning and synaptic plasticity. Biotech J.

[16] Ohshima T, Ogura H, Tomizawa K, Hayashi K, Suzuki H, Saito T, Kamei H, Nishi A, Bibb JA, Hisanaga S, Matsui H, Mikoshiba K (2005). Impairment of hippocampal long-term depression and defective spatial learning and memory in p35–/– mice. J Neurochem.

[17] Hayashi S, McMahon AP (2000). Efficient recombination in diverse tissues by a tamoxifen-inducible form of cre: A tool for temporally regulated gene activation/inactivation in the mouse. Dev Biol.

[18] Pi HJ, Otmakhov N, Lemelin D, De Koninck P, Lisman J (2010). Autonomous CaMKII can promote either long-term potentiation or long-term depression, depending on the state of T305/T306 phosphorylation. J Neurosci.

[19] Brigman JL, Wright T, Talani G, Prasad-Mulcare S, Jinde S, Seabold GK, Mathur P, Davis MI, Bock R, Gustin RM, Colbran RJ, Alvarez VA, Nakazawa K, Delpire E, Lovinger DM, Holmes A (2010). Loss of GluN2B-containing NMDA receptors in CA1hippocampus and cortex impairs long-term depression, reduces dendritic spine density, and disrupts learning. J Neurosci.

[20] Unoki T, Matsuda S, Kakegawa W, Van NT, Kohda K, Suzuki A, Funakoshi Y, Hasegawa H, Yuzaki M, Kanaho Y (2012). NMDA receptor-mediated PIP5K activation to produce PI(4,5)P_2_ is essential for AMPA receptor endocytosis during LTD. Neuron.

[21] Ngyuen C, Nishi A, Kansy JW, Fernandez J, Hayashi K, Gillardon F, Hemmings HC, Nairn AC, Bibb JA (2007). Regulation of protein phosphatase inhibitor-1 by cyclin-dependent kinase 5. J Biol Chem.

[22] Pontrello CG, Sun MY, Lin A, Fiacco TA, DeFea KA, Ethell IM (2012). Cofilin under control of β-arrestin-2 in NMDA-dependent dendritic spine plasticity, long-term depression (LTD), and learning. Proc Natl Acad Sci U S A.

[23] Gillardon F, Steinlein P, Burger E, Hildebrandt T, Gerner C (2005). Phosphoproteome and transcriptome analysis of the neuronal response to a CDK5 inhibitor. Proteomics.

[24] Seeburg DP, Feliu-Mojer M, Gaiottino J, Pak DT, Sheng M (2008). Critical role of CDK5 and Polo-like kinase 2 in homeostatic synaptic plasticity during elevated activity. Neuron.

[25] Sung JY, Engman O, Teylan MA, Nairn AC, Greengard P, Kim Y (2008). WAVE1 controls neuronal activity-induced mitochondrial distribution in dendritic spines. Proc Natl Acad Sci U S A.

[26] Cheung ZH, Chin WH, Chen Y, Ng YP, Ip NY (2007). Cdk5 is involved in BDNF-stimulated dendritic growth in hippocampal neurons. PLoS Biol.

[27] Deininger K, Eder M, Kramer ER, Zieglgänsberger W, Dodt HU, Dornmair K, Colicelli J, Klein R (2008). The Rab5 guanylate exchange factor Rin1 regulates endocytosis of the EphA4 receptor in mature excitatory neurons. Proc Natl Acad Sci U S A.

[28] Woolley CS, Weiland NG, Schwartzkroin PA (1997). Estradiol increases the sensitivity of hippocampal CA1 pyramidal cells to NMDA receptor-mediated synaptic input: correlation with dendritic spine density. J Neurosci.

[29] Mita N, He X, Sasamoto K, McEwen BS, Mishiba T, Ohshima T: **Cyclin-dependent kinase 5 regulates dendritic spine formation and maintenance of cortical neuron in the mouse brain.** *Cereb Cortex*, in press. 10.1093/cercor/bhu264 25404468

[30] Zamanillo D, Sprengel R, Hvalby O, Jensen V, Burnashev N, Rozov A, Kaiser KM, Köster HJ, Borchardt T, Worley P, Lübke J, Frotscher M, Kelly PH, Sommer B, Andersen P, Seeburg PH, Sakmann B (1999). Importance of AMPA receptors for hippocampal synaptic plasticity but not for spatial learning. Science.

[31] Lee HK, Barbarosie M, Kameyama K, Bear MF, Huganir RL (2000). Regulation of distinct AMPA receptor phosphorylation sites during bidirectional synaptic plasticity. Nature.

[32] Boehm J, Malinow R (2005). AMPA receptor phosphorylation during synaptic plasticity. Biochem Soc Trans.

[33] Miller SG, Kennedy MB (1986). Regulation of brain type II Ca2+/calmodulin-dependent protein kinase by autophosphorylation: a Ca2+-triggered molecular switch. Cell.

[34] Soderling TR, Fukunaga K, Rich DP, Fong YL, Smith K, Colbran RJ (1990). Regulation of brain Ca2+/calmodulin-dependent protein kinase II. Adv Second Messenger Phosphoprotein Res.

[35] Dhavan R, Greer PL, Morabito MA, Orlando LR, Tsai LH (2002). The cyclin-dependent kinase 5 activators p35 and p39 interact with the alpha-subunit of Ca2+/calmodulin-dependent protein kinase II and alpha-actinin-1 in a calcium-dependent manner. J Neurosci.

[36] Hosokawa T, Saito T, Asada A, Ohshima T, Itakura M, Takahashi M, Fukunaga K, Hisanaga S (2006). Enhanced activation of Ca2+/calmodulin-dependent protein kinase II upon downregulation of cyclin-dependent kinase 5-p35. J Neurosci Res.

[37] Wei FY, Tomizawa K, Ohshima T, Asada A, Saito T, Nguyen C, Bibb JA, Ishiguro K, Kulkarni AB, Pant HC, Mikoshiba K, Matsui H, Hisanaga S (2005). Control of cyclin-dependent kinase 5 (Cdk5) activity by glutamatergic regulation of p35 stability. J Neurochem.

[38] Hashimoto Y, Schworer CM, Colbran RJ, Soderling TR (1987). Autophosphorylation of Ca2+/calmodulin-dependent protein kinase II. Effects on total and Ca2+-independent activities and kinetic parameters. J Biol Chem.

[39] Abdirahman MJ, Fenton J, Robertson SD, Török K (2009). Time-dependent Autoinactivation of Phospho-Thr286-αCa2+/Calmodulin-dependent Protein Kinase II. J Biol Chem.

[40] Coultrap SJ, Freund RK, O’Leary H, Sanderson JL, Roche KW, Dell’acqua ML, Bayer KU (2014). Autonomous CaMKII mediates both LTP and LTD using a mechanism for differential substrate site selection. Cell Rep.

[41] Cruz JC, Tseng HC, Goldman JA, Shih H, Tsai LH (2003). Aberrant Cdk5 activation by p25 triggers pathological events leading to neurodegeneration and neurofibrillary tangles. Neuron.

[42] Su SC, Rudenko A, Cho S, Tsai LH (2013). Forebrain-specific deletion of Cdk5 in pyramidal neurons results in mania-like behavior and cognitive impairment. Neurbiol Learn Mem.

[43] He X, Wada S, Ishizeki M, Araki Y, Mita N, Ogura H, Abe M, Yamazaki M, Sakimura K, Mikoshiba K, Inoue T, Ohshima T: **Cdk5/p35 is required for motor coordination and cerebellar plasticity.** *J Neurochem*, in press. 10.1111/jnc.12756 24802945

[44] Ohshima T, Hirasawa M, Tabata H, Mutoh T, Adachi T, Suzuki H, Saruta K, Iwasato T, Itohara S, Hashimoto M, Nakajima K, Ogawa M, Kulkarni AB, Mikoshiba K (2007). Cdk5 is required for multipolar-to-bipolar transition during radial neuronal migration and proper dendrite development of pyramidal neurons in the cerebral cortex. Development.

[45] Tanaka M, Shih PY, Gomi H, Yoshida T, Nakai J, Ando R, Furuichi T, Mikoshiba K, Semyanov A, Itohara S (2013). Astrocytic Ca2+ signals are required for the functional integrity of tripartite synapses. Mol Brain.

[46] Sadakata T, Shinoda Y, Oka M, Sekine Y, Sato Y, Saruta C, Miwa H, Tanaka M, Itohara S, Furuichi T (2012). Reduced axonal localization of a Caps2 splice variant impairs axonal release of BDNF and causes autistic-like behavior in mice. Proc Natl Acad Sci U S A.

[47] Haws ME, Kaeser PS, Jarvis DL, Sudhof TC, Powell CM (2012). Region-specific deletions of RIM1 reproduce a subset of global RIM1α-/- phenotypes. Genes Brain Behav.

