# Supplementary material for: Cdk5/p35 functions as a crucial regulator of spatial learning and memory
Source: Mol Brain. 2014 Nov 18;7:82. doi: 10.1186/s13041-014-0082-x (PMC4239319; doi:10.1186/s13041-014-0082-x)
Supplement: Additional file 1: Figure S1. — No changes of Cdk5 protein levels in hippocampal homogenates from CreER-p35 cKO mice. Western blot analysis of Cdk5 protein in hippocampal homogenates from control and CreER-p35 cKO mice (p35 cKO) with anti-Cdk5 and anti-actin antibodies (A). No difference was found in Cdk5 protein levels among two genotypes (B). n = 4. [file 13041_2014_82_MOESM1_ESM.doc]

**Additional file 1.**


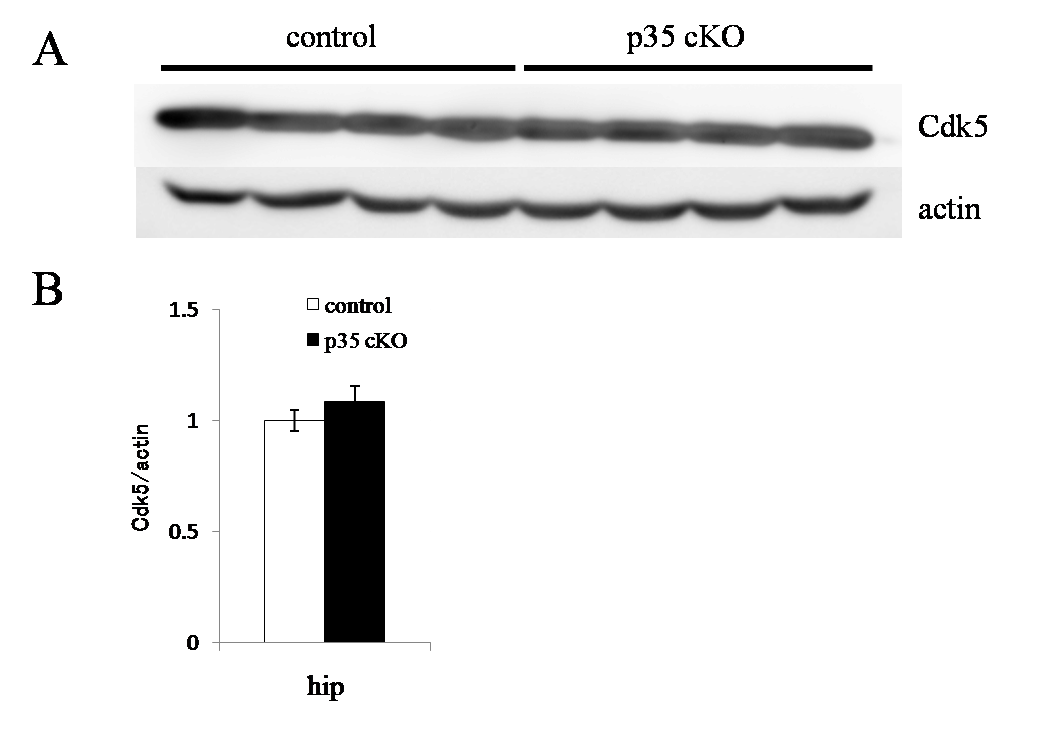


**Supplemental Figure 1. No changes of Cdk5 protein levels in hippocampal homogenates from CreER-p35 cKO mice.** Western blot analysis of Cdk5 protein in hippocampal homogenates from control and CreER-p35 cKO mice (p35 cKO) with anti-Cdk5 and anti-actin antibodies (A). No difference was found in Cdk5 protein levels among two genotypes (B). n=4.
