# Supplementary material for: Cdk5/p35 functions as a crucial regulator of spatial learning and memory
Source: Mol Brain. 2014 Nov 18;7:82. doi: 10.1186/s13041-014-0082-x (PMC4239319; doi:10.1186/s13041-014-0082-x)
Supplement: Additional file 2: Figure S2. — NMDA receptor-dependent LTD induction in the presence of tPDC. LTD induced by LFS in the presence of 300 μM tPDC was significantly blocked by application of D-AP5.Hippocampal slices from wild-type animals (C57BL/6 J) were used. [file 13041_2014_82_MOESM2_ESM.doc]

**Additional file 2.**


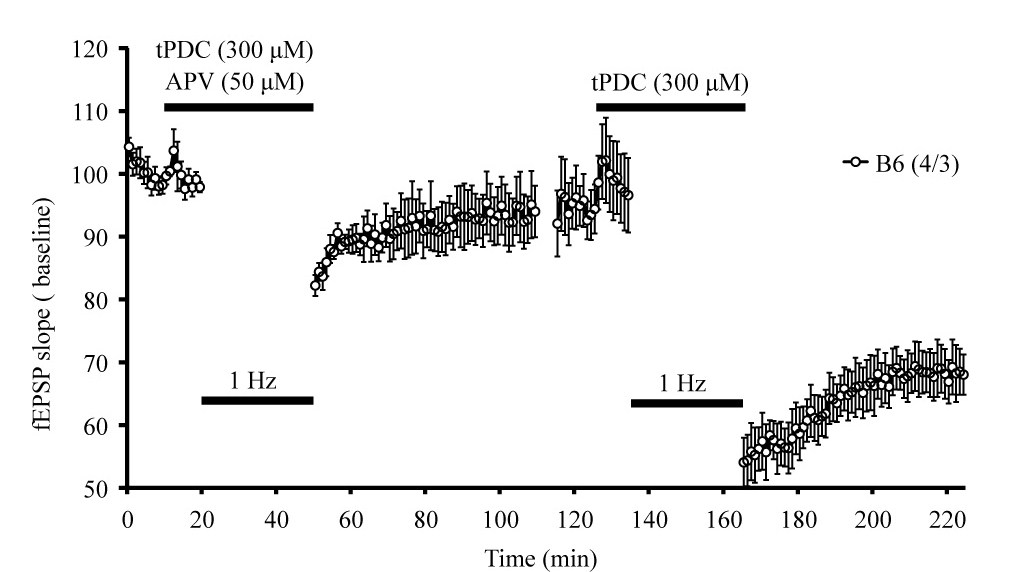


**Supplemental Figure 2. NMDA receptor-dependent LTD induction in the presence of tPDC.** LTD induced by LFS in the presence of 300 µM tPDC was significantly blocked by application of D-AP5.Hippocampal slices from wild-type animals (C57BL/6J) were used.
