# Supplementary material for: Cdk5/p35 functions as a crucial regulator of spatial learning and memory
Source: Mol Brain. 2014 Nov 18;7:82. doi: 10.1186/s13041-014-0082-x (PMC4239319; doi:10.1186/s13041-014-0082-x)
Supplement: Additional file 3: Figure S3. — No neuro-inflammation in hippocampal CA1 in CreER-p35 cKO mice. Immunostaining of hippocampal sections of control and CreER-p35 cKO mice (p35 cKO) with Iba1 to observe activated microglia (A), with GFAP to observe activated astrocytes (B) and with DAPI. [file 13041_2014_82_MOESM3_ESM.doc]

**Additional file 3.**


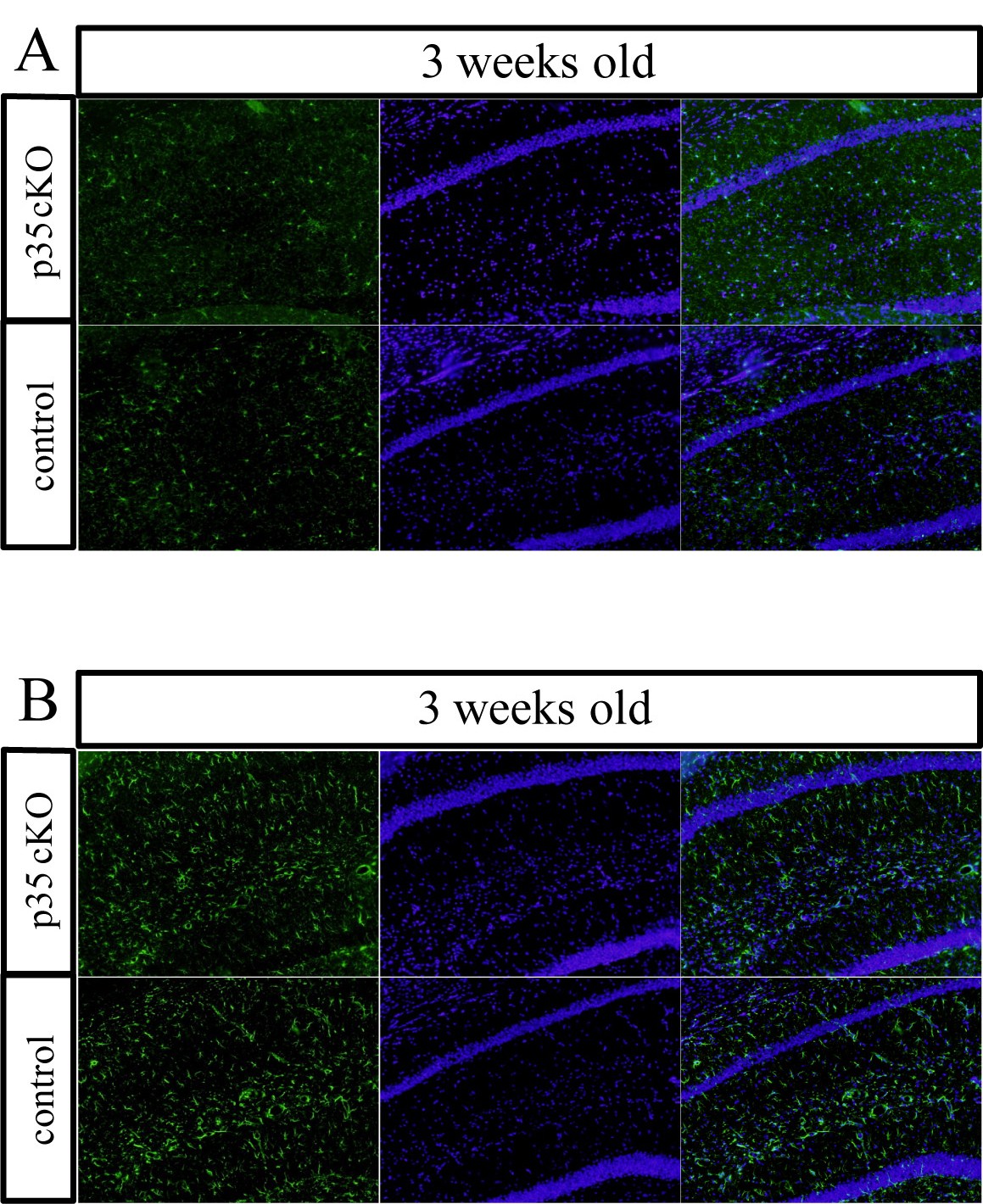


**Supplemental Figure 3. No neuro-inflammation in hippocampal CA1 in CreER-p35 cKO mice.**

Immunostaining of hippocampal sections of control and CreER-p35 cKO mice (p35 cKO) with Iba1 to observe activated microglia (A), with GFAP to observe activated astrocytes (B) and with DAPI.
